# Ground-penetrating radar datasets for non-destructive geometry evaluation of concrete step specimens with embedded elements^✰^

**DOI:** 10.1016/j.dib.2026.113103

**Published:** 2026-07-24

**Authors:** Maria Grohmann, Jens Wöstmann, Stefan Maack, Ernst Niederleithinger

**Affiliations:** Federal Institute for Materials Research and Testing (BAM), Unter den Eichen 87, 12205 Berlin, Germany

**Keywords:** Civil engineering, Concrete structures, Ground-penetrating radar method, Reference Data

## Abstract

The datasets comprise raw data collected using ground-penetrating radar (GPR) in pulse-echo mode on three distinct concrete specimens. The specimens represent fundamental non-destructive testing (NDT) challenges in civil engineering, such as locating built-in elements and evaluating their dimensions to characterize both the internal and external geometry of concrete structures. Data acquisition was performed using two bistatic antennas with centre frequencies of 1.5 GHz and 2.6 GHz. Both antennas were mounted on a scanner system, enabling automated measurements with high positional accuracy and high spatial sampling density. During each measurement, only one antenna was active. This article provides a comprehensive description of the uploaded datasets, the design of the three concrete specimens, the technical specifications of the GPR equipment, and the data acquisition process. The raw GPR data are provided in a universally readable format and consist of the recorded time signals stored at each individual measurement point across the scanned area. The datasets are intended to support comparative studies in signal processing, imaging, and data interpretation, serving as a reference for various real-world NDT scenarios in civil engineering.

Specifications Table

**Table:** 

Subject	Engineering & Materials science
Specific subject area	Automated ground-penetrating radar measurements on three concrete specimens for diverse non-destructive testing (NDT) scenarios in civil engineering.
Type of data	Time series; Raw Data; Formatted Data
Data collection	The ground-penetrating radar (GPR) data were collected on three concrete specimens using an automated scanner system. Two specimens were produced at the Bundesanstalt für Materialforschung und -prüfung (Federal Institute for Materials Research and Testing, BAM) in Berlin. The third specimen was constructed at the Materials Testing Institute MPA in Stuttgart and is now located at BAM. Automated scanning system and scanner control: developed and manufactured by BAM GPR antennas (1.5 GHz und 2.6 GHz): manufactured by Geophysical Survey Systems, Inc. (GSSI) Radar system (SIR-20): manufactured by GSSI
Data source location	Institution: Bundesanstalt für Materialforschung und -prüfung, BAM City/Town/Region: Berlin Country: Germany
Data accessibility	Repository name: HARVARD Dataverse Data identification number: 10.7910/DVN/FCMUJQ Direct URL to data: https://doi.org/10.7910/DVN/FCMUJQ
Related research article	None

## 1 Value of the Data

• Ground-penetrating radar (GPR) measurements on concrete structures in real-world non-destructive testing (NDT) applications are influenced by application-specific conditions, including material properties, internal geometries, and measurement conditions, which can present challenges for data interpretation. The provided datasets include controlled laboratory measurements on three well-documented concrete specimens with known internal features and material properties, representing common scenarios encountered in NDT of concrete structures.
• The datasets can be used for different GPR evaluation tasks, depending on the concrete specimen. All three specimens can be used to study the detection and lateral and depth localization of rebars in concrete and to evaluate concrete thicknesses and a stepped back-wall geometry. Beyond rebars, specimens Pk266 and Pk401 allow detection and lateral and depth localization of additional embedded objects: tendon ducts at different depths in Pk266, and non-metallic rigid polystyrene foam cuboids at different embedment depths in Pk401.
• The datasets can be reused by researchers and practitioners to develop, test, and validate GPR data processing, imaging, and interpretation methods under well-defined boundary conditions.
• The datasets were acquired with high spatial accuracy, and all data acquisition parameters are fully documented to support comparative studies of different GPR data processing workflows and algorithms.
• The datasets can be used for studies on data-driven analysis methods, including machine learning approaches for NDT of concrete structures.
• The presented concrete specimens can serve as a reference for developing new experimental setups in GPR NDT research.
• The datasets can be used for training, education, and qualification of personnel involved in GPR-based inspection of concrete structures.

## 2 Background

This article builds upon the work of Maack et al. [1] and Grohmann et al. [2], in which ultrasonic reference datasets from the same concrete specimens investigated in this paper were published. The present data article extends this experimental framework by providing datasets acquired using GPR [3]. GPR is another widely used state-of-the-art NDT technique for concrete inspection [4]. In GPR measurements, electromagnetic pulses are sent into concrete components by a transmitting antenna [5]. Reflections occur at internal interfaces or embedded objects with contrasting electromagnetic properties and are recorded by a receiving antenna [5]. The recorded signals are then evaluated to analyse the internal structure of the investigated concrete components, for example to determine thicknesses and layer structures [6]. Further typical GPR applications are the localization of embedded elements, such as reinforcement and tendon ducts [7], as well as other objects, for example anchors, brackets, and wooden elements [5]. GPR is also used for the detection of defects and damage, such as voids and grouting defects in post-tensioned concrete members [8]. In addition, it can be used to investigate material properties, such as moisture and salt content [[9], [10], [11]].

For the development and evaluation of GPR data processing and interpretation methods, reference datasets collected under controlled laboratory conditions are required. Such datasets are particularly useful when the external and internal specimen geometry as well as the material parameters are documented, as in the GPR datasets presented in this article [Table 1, APPENDICES A - C]. Furthermore, GPR and ultrasonic measurements provide complementary information on investigated concrete structures [7], because the methods are sensitive to different material and structural properties. Therefore, by complementing the existing ultrasonic datasets [1,2] with GPR measurements, a combined experimental dataset is provided that can be used, for example, for developing data fusion approaches [12,13] as well as for comparative studies of different measurement techniques in NDT of concrete structures.

## 3 Data Description

**Fig. 1:**
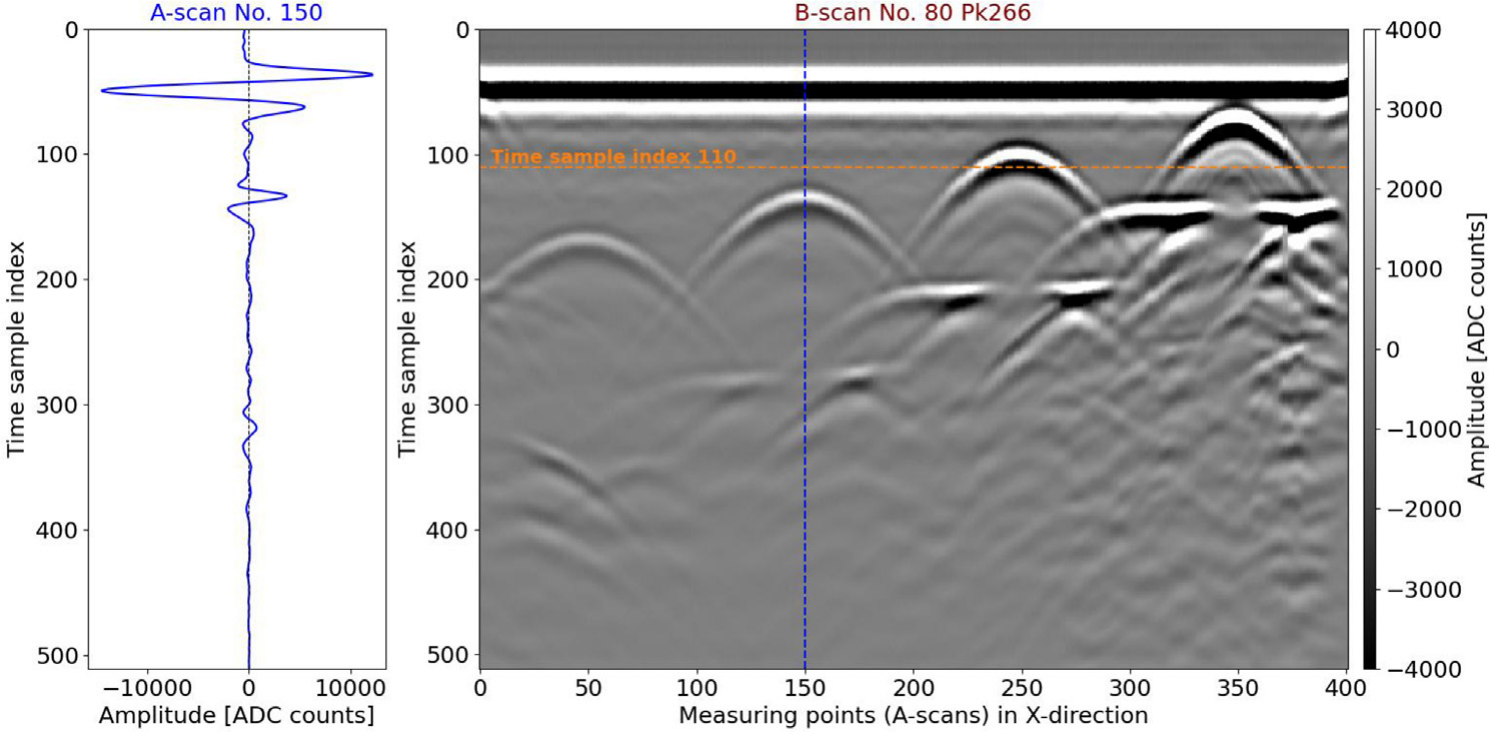
Overview of the designations of the data that can be derived from a line measurement (data from Pk266): left: time-signal at a single measurement point (A-scan); right: time-signals (A-scans) sampled along a measurement line (B-scan). The position of the C-scan at time sample index 110 (Fig. 2) is indicated by an orange dashed line.

**Fig. 2:**
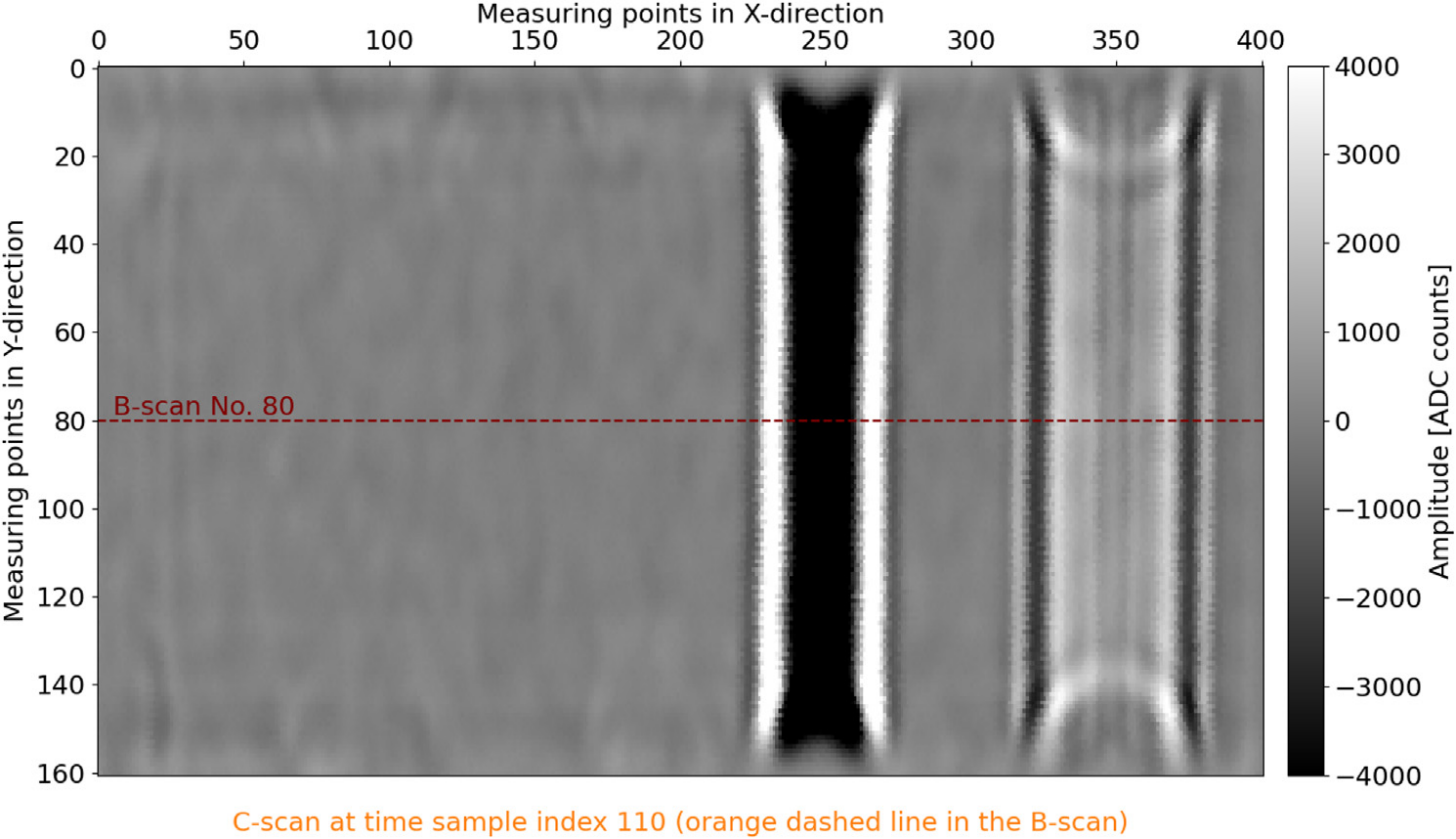
Depth slice through time (C-scan) from Pk266 at time sample 110. The position of the B-scan No. 80 (Fig. 1, right) is indicated by a red dashed line.

The GPR datasets are raw data collected on a defined measurement grid on the surface of three concrete specimens. The internal designations used at the Bundesanstalt für Materialforschung und -prüfung (Federal Institute for Materials Research and Testing, BAM) for the specimens are Pk050, Pk266 and Pk401 (‘Pk’ is a German abbreviation for ‘Prüfkörper’, English: ‘test specimen’). At each measurement point, a pulse-echo measurement was performed, with the transmitting and receiving GPR antennas integrated into a single antenna housing (bistatic configuration) (Fig. 10c) [5]. During data acquisition, an electromagnetic pulse was emitted into the specimen by the transmitting antenna, and the resulting reflected signals were recorded by the receiving antenna. The signal obtained from a single pulse-echo measurement is a time-domain signal, commonly referred to as an A-scan in GPR testing [5]. It displays the amplitude of the received electromagnetic signal as a function of two-way travel time. Fig. 1 (left) shows an exemplary A-scan of the data collected on Pk266. By recording A-scans at multiple points along a measurement line and color-coding the amplitude values, a two-dimensional (2D) profile, commonly referred to as a B-scan, can be generated. A B-scan represents the spatial variation of the reflected electromagnetic signal along the measurement line as a function of two-way travel time. Fig. 1 (right) illustrates an exemplary B-scan of Pk266, where the signal amplitude is displayed in grayscale. By acquiring multiple parallel B-scans over a defined measurement area, a three-dimensional (3D) dataset can be generated. From this 3D dataset horizontal slices at selected two-way travel times, commonly referred to as C-scans, can be extracted [5]. These C-scans provide planar views of structural features within the investigated medium (Fig. 2).

For the measurements on the concrete specimens Pk050, Pk266 and Pk401 two bistatic GPR antennas with centre frequencies of 1.5 GHz and 2.6 GHz were used (Fig. 10c). Each antenna was operated in two orientations: 0° and 90°, which define the direction of polarization. The two antenna orientations are described in more detail in section MEASUREMENT SETUP AND PROCESS (Fig. 11). The measurements were performed with an acquisition time of t = 15 ns, and each A-scan consists of 512 samples (see Table 2 in section MEASUREMENT SETUP AND PROCESS). This corresponds to a time sampling interval of Δt = 0.029354 ns and a sampling rate of f = 34.06 GHz. The data were stored by the radar system as 16-bit unsigned integers representing radar signal amplitudes in digital units (analog-to-digital conversion (ADC) counts). The corresponding Nyquist frequency is 17.03 GHz, which is well above the highest recorded frequency of 5 GHz (defined by the low-pass filter settings used for the 1.5 GHz and 2.6 GHz antennas, see Table 2 in section MEASUREMENT SETUP AND PROCESS). Therefore, the temporal sampling is sufficient to avoid undersampling of the recorded GPR frequency range.

**Fig. 3:**
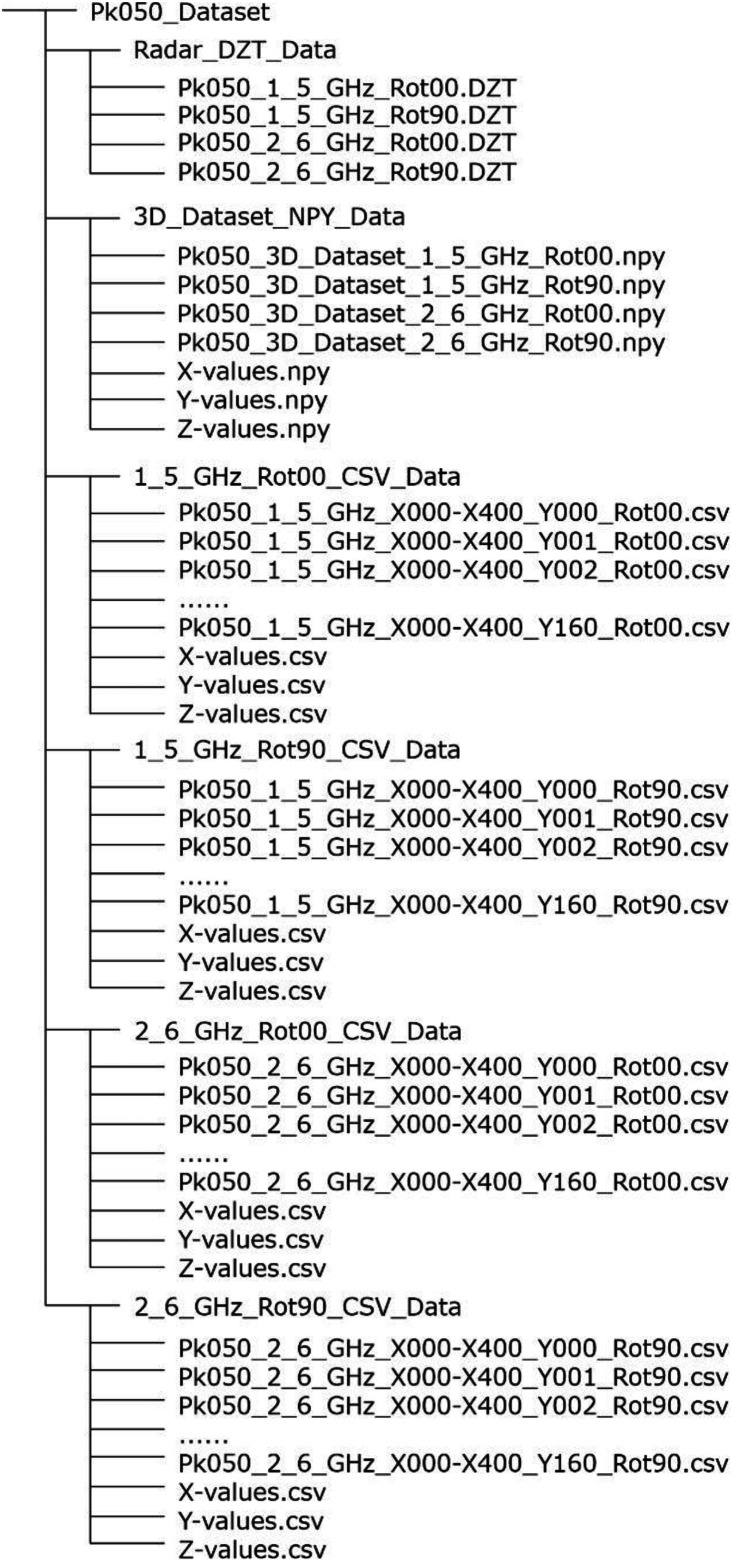
Folder structure and available data types for specimen Pk050; Pk266 and Pk401 follow the same structure.

The datasets include the native radar files (*.DZT) as recorded by the measurement system as well as formatted versions derived from these files. Formatting of the native *.DZT files was necessary because the radar system stores the recorded A-scans as one continuous sequence, independent of the measurement grid. The formatting steps are described in detail in section DATA FORMATTING WORKFLOW. The formatted datasets provide the measurement signals (time-domain signals) as well as geometrical information and are available in different formats and types. First, individual B-scans (which consist of a series of A-scans sampled consecutively along a measurement line, as shown in Fig. 1) are provided in the commonly accessible CSV format (∗.csv files) [14]. Second, the measurement data are made available in NumPy format (∗.npy files, Python version 3.11.7) in a 3D array. Fig. 3 illustrates the folder structure and the available data types, using specimen Pk050 as an example (Pk266 and Pk401 follow the same structure).

**Fig. 4:**
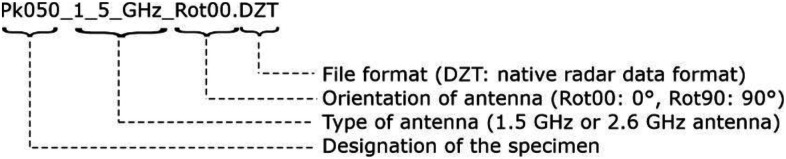
Exemplary description of the file names for the native radar files recorded by the measurement system (*.DZT).

**Fig. 5:**
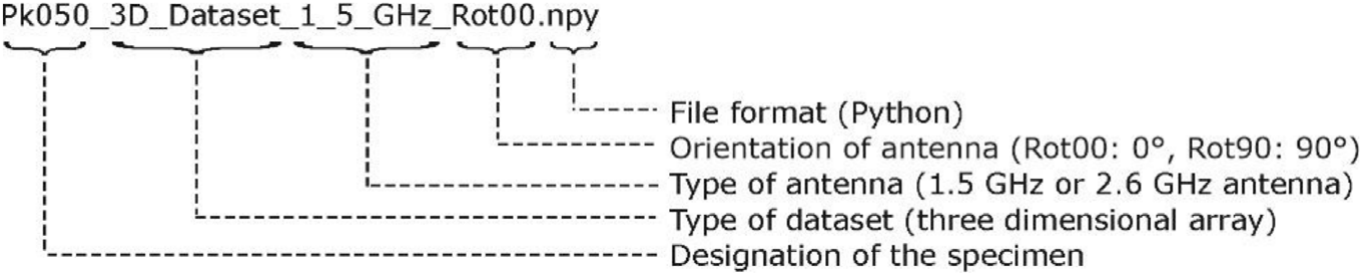
Exemplary description of the file names for the files stored in NumPy-format (*.npy).

The first subfolder, *Radar_DZT_Data*, includes the native radar files (*.DZT). These can be read in Python, for example using the readgssi package [15]. The designation for the *.DZT data is described in Fig. 4 exemplarily. The four datasets differ by antenna type (1.5 GHz or 2.6 GHz) and antenna orientation (Rot00 (0°) or Rot90 (90°)). The subfolder, *3D_Dataset_NPY_Data*, contains the 3D datasets in NumPy format (*.npy). They can be loaded in Python using np.load("path/to/file.npy"). In addition to the 3D datasets, the subfolder provides three vectors: the measurement coordinates in the X and Y directions (X-values and Y-values, in mm) and the sample time values (Z-values, in ns). The X- und Y- axes of the measurement plane of Pk050 are shown in APPENDIX A. The designation for the 3D datasets is explained in Fig. 5 exemplarily.

**Fig. 6:**
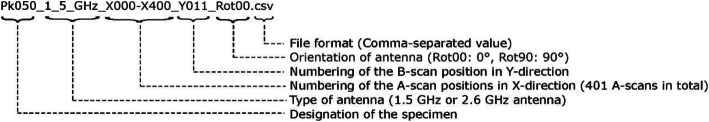
Exemplary description of the file names for the files stored in CSV-format (*.csv).

The data tree (Fig. 3) further includes four additional subfolders that contain two-dimensional (2D) B-scan datasets in CSV format (*.csv files). The subfolder names indicate the antenna type and the antenna orientation. In contrast to the 3D datasets in NumPy format, each CSV file stores an individual B-scan along the X-axis of the measurement area. Each subfolder also includes three vectors: X-values and Y-values (in mm) for the measurement positions, and Z-values (in ns) for the sample times. The naming scheme for the 2D datasets is illustrated in Fig. 6 exemplarily. The A-scan labelled X000 is located at the origin of the coordinate system (X/Y = 0 mm), which is marked in the technical drawings in the APPENDICES and in Fig. 13 by a black circled cross. The B-scan labelled Y000, which contains the A-scans X000 – X400, is located at Y = 0 mm. The locations of B-scan Y000 and A-scans X000 and X400 are shown in Fig. 13 in section DATA FORMATTING WORKFLOW.

## 4 Experimental Design, Materials and Methods

### 4.1 Concrete specimens PK050, PK266, AND PK401

The concrete specimens Pk050, Pk266, and Pk401 were produced as part of the research project „FOR 384: Zerstörungsfreie Strukturbestimmung von Betonbauteilen mit akustischen und elektromagnetischen Echo-Verfahren“ [16]. This project was funded by the German Research Foundation (Deutsche Forschungsgemeinschaft DFG) in the years 2000 to 2007 (grant number: 5466784). The specimens Pk050 and Pk266 were produced at BAM in Berlin, whereas Pk401 was manufactured at the Materials Testing Institute (MPA) in Stuttgart and is now also owned by BAM. Specimen Pk050 and Pk266 were stored under dry indoor laboratory conditions before the GPR measurements. Specimen Pk401 was stored outdoors, but under protected and covered conditions. At the time of the GPR scans, the specimens were approximately 25 years old.

All three specimens are step-shaped concrete blocks with identical external geometry and dimensions. Each specimen measures 2000 mm in length and 800 mm in width, and has a stepped profile with four thickness levels, each 500 mm long. While the outer geometry is identical, the specimens differ in their internal structure. In addition, the specimens were manufactured under controlled laboratory conditions. All sides of the three specimens exhibit production-related surface imperfections of approximately ±1 mm. Due to the controlled manufacturing process, the positional deviations of the embedded elements are assumed to be in the range of a few millimetres. These deviations are assumed to be smaller than typical tolerances specified for construction-site conditions. National and international standards, guidelines, and model codes for concrete structures [[17], [18], [19]] commonly allow deviations for embedded components and reinforcement placement in the range of several millimetres to centimetres.

#### 4.1.1 Specimen PK050

**Fig. 7:**
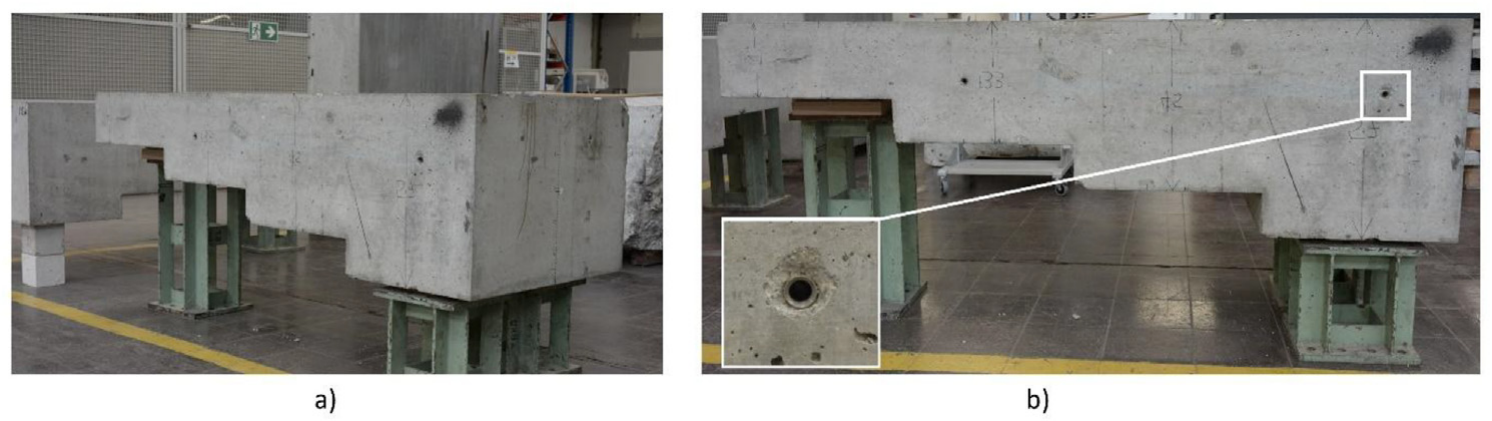
Concrete specimen Pk050: a) 3D perspective and b) lateral view with a highlighted impact anchor.

Pk050 is a plain concrete step specimen and has no embedded elements (Fig. 7). Only near-surface steel bars (diameter: 10 mm) were installed at a mounting depth of 30 mm to prevent cracking. In addition, four galvanized M16 impact anchors were installed on the sides (Fig. 7). A technical drawing of the specimen is provided in APPENDIX A.

The concrete mixture (e.g. water-cement ratio, air void content and cement type) and the aggregate grading curve (sieve line) are described in detail by Maack et al. [1] (see Table 2 and Fig. 9 in [1]) and are summarized in Table 1. The maximum aggregate size is 16 mm. The specimen surface exhibits a localized area of damage, where concrete spalling with a maximum depth of approximately 10 mm occurred (see Fig. 11 in Maack et al. [1]). Further details of the specimen, including a 3D view and information on geometric dimensions and structure, can be found in Maack et al. [1].

**Table 1:** Material parameters of specimens Pk050, Pk266 [1] and Pk401 [20].

Material parameter	Specific value Pk266 & Pk050	Specific value Pk401
Water-cement ratio (w/c)	0.47	0.47
Water (w)	193 kg/m³	190 kg/m³
Cement (c)	411 kg/m³	404 kg/m³
Consistency	KR, regular consistency	KR, regular consistency
Air void content	1 %	1.5 %
Concrete aggregate (total)	1762 kg/m³	1734.2 kg/m³
Grading curve	A/B 16	A/B 16
Cement type and strength class	CEM I 32.5 R	CEM I 32.5 R
Type of aggregate	Gravel sand	n.a.
Bulk density hardened concrete	2383 kg/m³	n.a.
Compressive strength	48.7 N/mm²	n.a.

#### 4.1.2 Specimen PK266

**Fig. 8:**
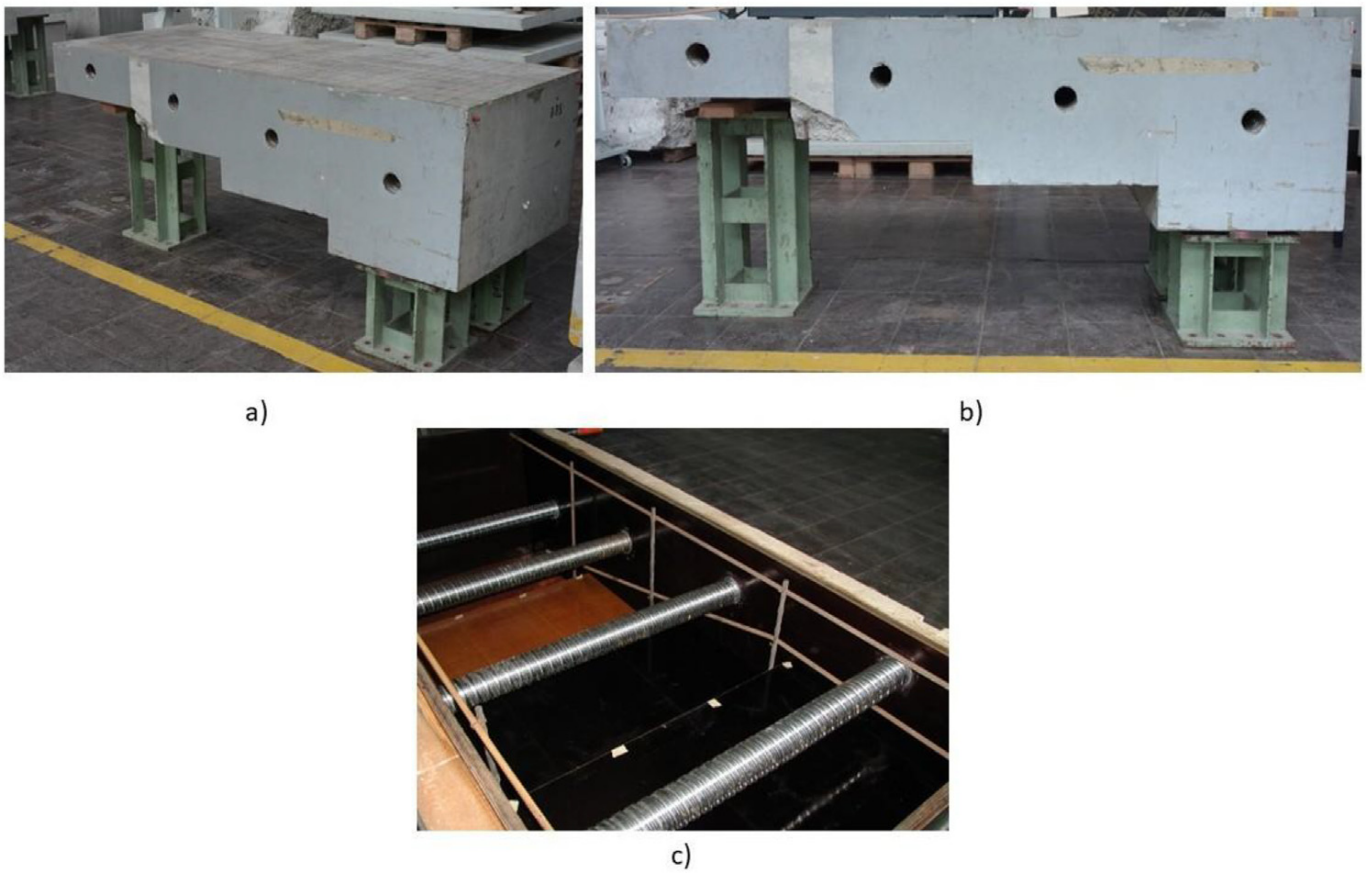
Concrete specimen Pk266: a) 3D perspective, b) lateral view and c) formwork with four embedded tendon ducts before casting.

Pk266 was produced using the same concrete mixture as Pk050 (Table 1). The key difference is that Pk266 contains four embedded tendon ducts (Fig. 8). The tendon ducts are made of sheet steel with a thickness of 0.4 mm and are corrugated at an angle (see Fig. 14 in Maack et al. [1]). Each duct has a circular cross section with an inner diameter of 60 mm and an outer diameter of 67 mm (see Fig. 14 in Maack et al. [1]) and is positioned within one of the four thickness sections. As with Pk050, only light reinforcement was used at the specimen edges to maintain structural integrity (Fig. 8c), with a reinforcement geometry identical to that of Pk050. Unlike Pk050, Pk266 does not include the four impact anchors. A detailed technical drawing of the specimen is provided in APPENDIX B. Maack et al. [1] provide further geometric and structural details of the specimen, including a 3D view (see, e.g., Table 4 and Figs. 12 and 13 in Maack et al. [1]).

#### 4.1.3 Specimen PK401

**Fig. 9:**
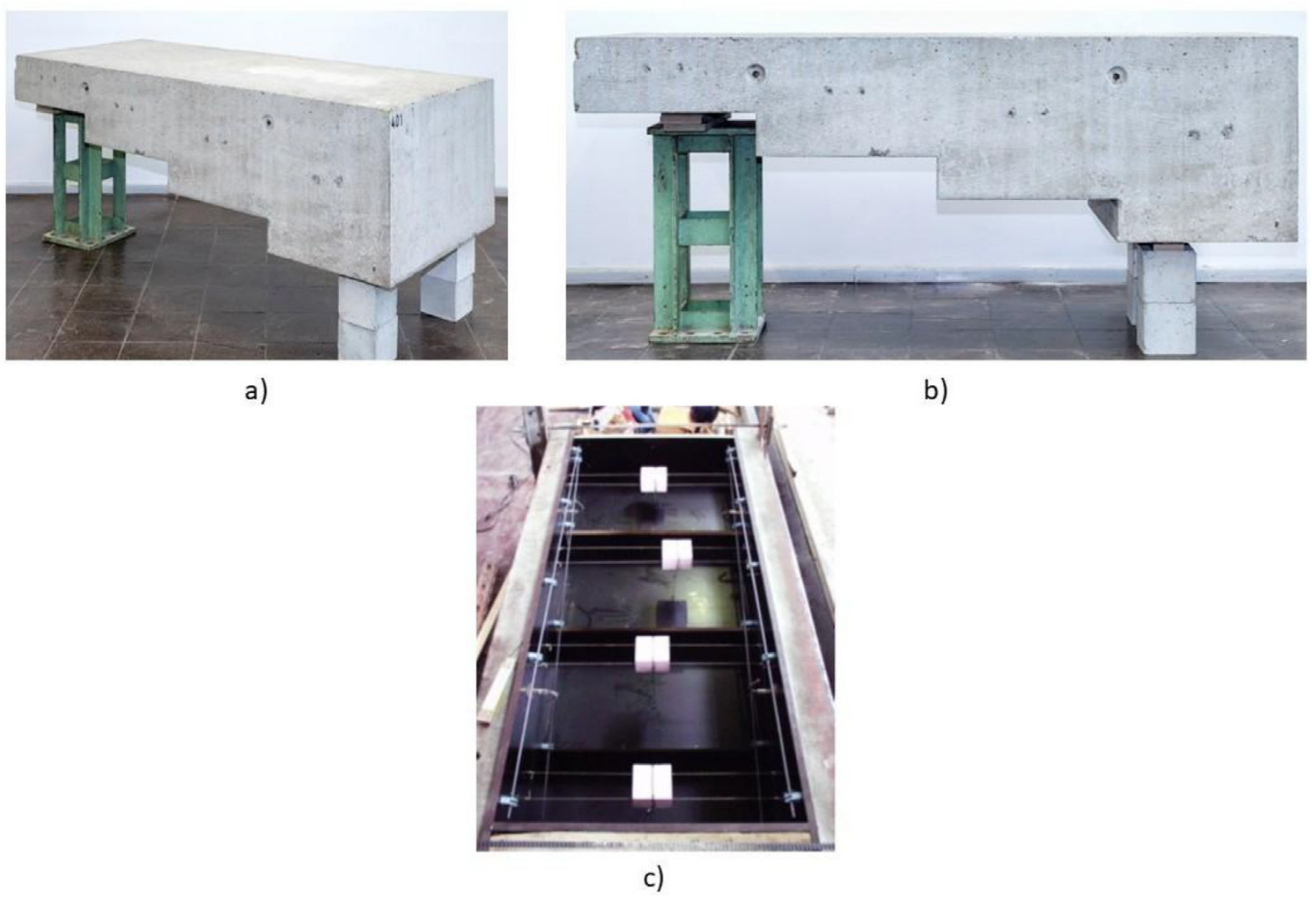
Concrete specimen Pk401: a) 3D perspective, b) lateral view and c) formwork with four embedded rigid polystyrene foam cuboids before casting (Fig. 9c was reproduced from Beutel et al. [20]).

Pk401 was designed following the same concept as Pk050 and Pk266 (similar size and four-step thickness profile) (Fig. 9). It was made of a similar concrete mixture (standard C30/37-class concrete with 16 mm aggregate) [2]. The available material parameters for Pk401 are listed in Table 1. The type of aggregate, bulk density, and compressive strength were not available for Pk401 and are therefore marked as n.a. in Table 1. This should be considered when these parameters are relevant for further analysis or modelling.

In contrast to Pk266, which contains tendon ducts, Pk401 incorporates four rigid polystyrene foam cuboids embedded in each of the four thickness levels (Fig. 9c). Each cuboid has dimensions of 120 × 120 × 60 mm (APPENDIX C). To prevent cracking, steel reinforcement was installed along the specimen edges (Fig. 9c, APPENDIX C). In Fig. 10 of Grohmann et al. [2] the reinforcement is highlighted in blue in the 3D model of Pk401.

**Fig. 10:**
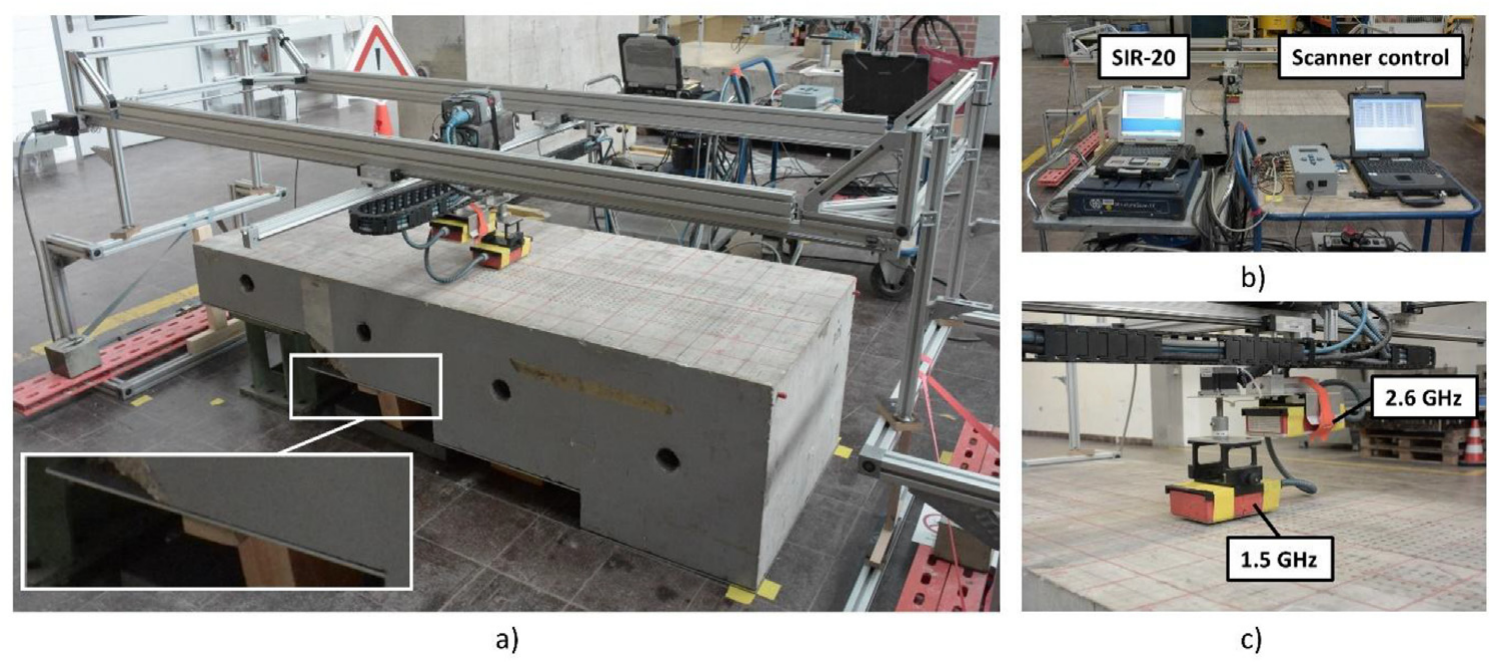
GPR measurement equipment: a) automated scanner system over specimen Pk266, carrying two GPR antennas; aluminium plates were attached to the lower edge of the specimen b) radar system SIR-20 and scanner control; c) GSSI 1.5 GHz and 2.6 GHz antennas (only the lower antenna was active during the measurements).

After casting, the surface of Pk401 was lightly sanded, resulting in a slightly rough surface (see Fig. 9a in Grohmann et al. [2]). Furthermore, the specimen’s surface exhibits partial spalling along the edges (Fig. 9c in Grohmann et al. [2]). Upon delivery from MPA Stuttgart to BAM, four previously undocumented surface boreholes were present (see Fig. 8 in Grohmann et al. [2]). These boreholes were filled at BAM using a cement-based grouting mortar (CEM I 42,5 R (ep)) [2].

Further construction details as well as a 3D model of the specimen can be found in Grohmann et al. [2]. A technical drawing of Pk401 is included in APPENDIX C. The reinforcement details of Pk401 shown in the technical drawing are approximate values, derived from GPR measurements as reported in Grohmann et al. [2]. Only for the reinforcement visible on the outer sides of Pk401 (marked blue and black in APPENDIX C and exemplarily shown in Fig. 9b in Grohmann et al. [2]) an exact diameter could be specified. Furthermore, a detailed verification of the positions of the rigid polystyrene foam cuboids as outlined in the construction plans of MPA Stuttgart was not technically feasible at BAM [2]. Small positional deviations may have occurred during concreting [2]. However, these deviations are expected to be minor because the embedded elements were rigidly fixed in place before casting (Fig. 9c).

### 4.2 Ground-Penetrating Radar Measurement Equipment

The GPR measurements were performed using the radar system SIR-20 manufactured by Geophysical Survey Systems, Inc. (GSSI) [21] (Fig. 10b), which was connected to one of the two bistatic GPR antennas used (GSSI 1.5 GHz or GSSI 2.6 GHz antenna [22]) (Fig. 10c). The two antenna centre frequencies were employed to obtain datasets with different penetration depths and spatial resolutions. The SIR-20 system controlled both pulse generation and the data acquisition chain, which included signal amplification, sampling, and ADC. During data acquisition, manufacturer-recommended filtering was applied by the SIR-20 system to the recorded signals. This included a linear time gain of 2 dB for the 1.5 GHz antenna and 6 dB for the 2.6 GHz antenna (Table 2). For both antennas, an IIR filter with a cut-off frequency of 10 MHz was applied. This was followed by FIR filtering with a high-pass cut-off frequency of 400 MHz and a low-pass cut-off frequency of 5000 MHz (Table 2).

The two GPR antennas were mounted on an automated scanner (custom-built at BAM) (Fig. 10a), which moved the antennas across the specimen surface on a defined measurement grid. During the measurements, only the lower antenna was active at a time (both antennas were swapped between the measurements). Two rotary encoders on the scanner axes were used for position tracking, and the scanner control (developed at BAM) (Fig. 10b) provided, among other functions, time-based trigger signals for the SIR-20 system to initiate data acquisition. In addition, aluminium plates (thickness: 5 mm) were attached to the lower edge of the stepped concrete specimens (Fig. 10a) to provide a well-defined, highly reflective boundary in the recorded GPR data. The aluminium plates generate clear back-wall reflections that can serve as reference events for estimating an apparent electromagnetic wave velocity $v_{app}$, apparent relative permittivity $\varepsilon_{r,app}$, and time-zero correction $t_{0}$ (see Table 4, section REFERENCE INFORMATION FOR DATA USE). The time-zero correction $t_{0}$ accounts for system-related delays and defines the effective start time of the recorded GPR signal.

### 4.3 Measurement Setup and Process

**Fig. 11:**
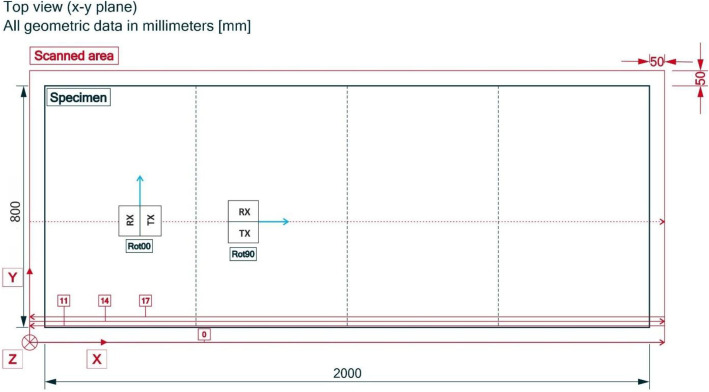
Overview of the scanned area with the applied antenna orientations (Rot00 and Rot90). Measurement lines No. 0, No. 11, No. 14, and No. 17 are highlighted as examples. TX denotes the transmitting antenna and RX the receiving antenna. The blue arrow indicates the direction of polarization.

To acquire the GPR data, an automated scanner system (Fig. 10a) was used to scan the surface of each concrete specimen in a meandering pattern along a predefined measurement grid. The scanned area was defined with a margin of 50 mm from all specimen edges (marked red in Fig. 11). Table 2 summarizes the measurement parameters used. The GPR antenna was moved along the X-direction, with a measurement point spacing of 2.5 mm in the X-direction and 5 mm in the Y-direction (Table 2). The origin of the measurement coordinate system is marked by a red circled cross in Fig. 11. It is located at the thin side of the stepped specimens (at the side with the smallest concrete thickness). Scan lines No. 0, No. 11, No. 14, and No. 17 are shown as examples by red lines with their respective scan directions. Furthermore, the GPR antenna was positioned at a constant distance of 5 mm from the concrete surface and moved continuously at a velocity of 0.55 m/s. While scanning, the antenna transmitted electromagnetic pulses and recorded the reflected signals. The acquisition time was 15 ns, and each A-scan consists of 512 samples (see Table 2). Fig. 11 illustrates the two different antenna orientations (TX: transmitting antenna and RX: receiving antenna) used which were denoted in the datasets with Rot00 and Rot90. For the Rot00 orientation the polarization of the antenna (blue arrow in Fig. 11) is aligned along the Y-direction, whereas for the orientation Rot90 the polarization of the antenna is aligned with the measurement direction (X-direction). These two orientations were used because the antennas exhibit direction-dependent characteristics with respect to the emitted electromagnetic field. The center-to-center spacing between the transmitting antenna (TX) and receiving antenna (RX) is 48 mm for the 1.5 GHz antenna and 40 mm for the 2.6 GHz antenna.

**Table 2:** Measurement parameters and data acquisition settings for Pk050, Pk266 and Pk401.

Parameter	Values	
Antenna type [GHz]	1.5	2.6
Measurement frequency [GHz]	1.5	2.6
Antenna orientation []	0° (Rot00) & 90° (Rot90) 2100×900 2.5 × 5 841 181 4 2 15 512	
Size scanned area [X x Y] [mm]		
Grid spacing scanned area [X x Y] [mm]		
Number of measurement points X-direction []		
Number of scan lines Y-direction []		
Sampling density X-direction [Samples/cm]		
Sampling density Y-direction [Samples/cm]		
Acquisition time [ns]		
Time samples (per A-scan) []		
IIR Filter cut-off frequency [MHz]	10	
FIR Filter high-pass cut-off frequency [MHz]	400	
FIR Filter low-pass cut-off frequency [MHz]	5000	
Linear time gain [dB]	2	6

The positioning accuracy of the scanner system (Fig. 10a) is approximately 0.14 mm. Therefore, deviations from the planned measurement positions are small compared to the measurement point spacing (2.5 mm and 5 mm). This precise positioning of the GPR antenna is a major advantage over manual GPR measurements. For manually acquired GPR data, a directly comparable value for positioning accuracy is difficult to specify, because it depends, for example, on operator handling and surface conditions. However, the positioning uncertainty is expected to be larger than for the automated scanner system. In addition, the automated scanner provides reproducible positioning, whereas repeated manual GPR measurements show positional shifts in the millimeter range in practice. An acquisition-related offset was observed during measurements with the scanner system. This offset occurred between adjacent scan lines and was caused by the delay between the trigger signal from the scanner control and the actual start of the data acquisition in the SIR-20 radar system. This issue is described in detail in the section LIMITATIONS. The offset was successfully corrected during data formatting (section DATA FORMATTING WORKFLOW), and the remaining scan-line misalignment is expected to be smaller than half of the original trace spacing (< 1.25 mm). This ensures a consistent assignment of each A-scan to its corresponding X/Y coordinate in the final dataset.

### 4.4 Data Formatting Workflow

**Fig. 12:**
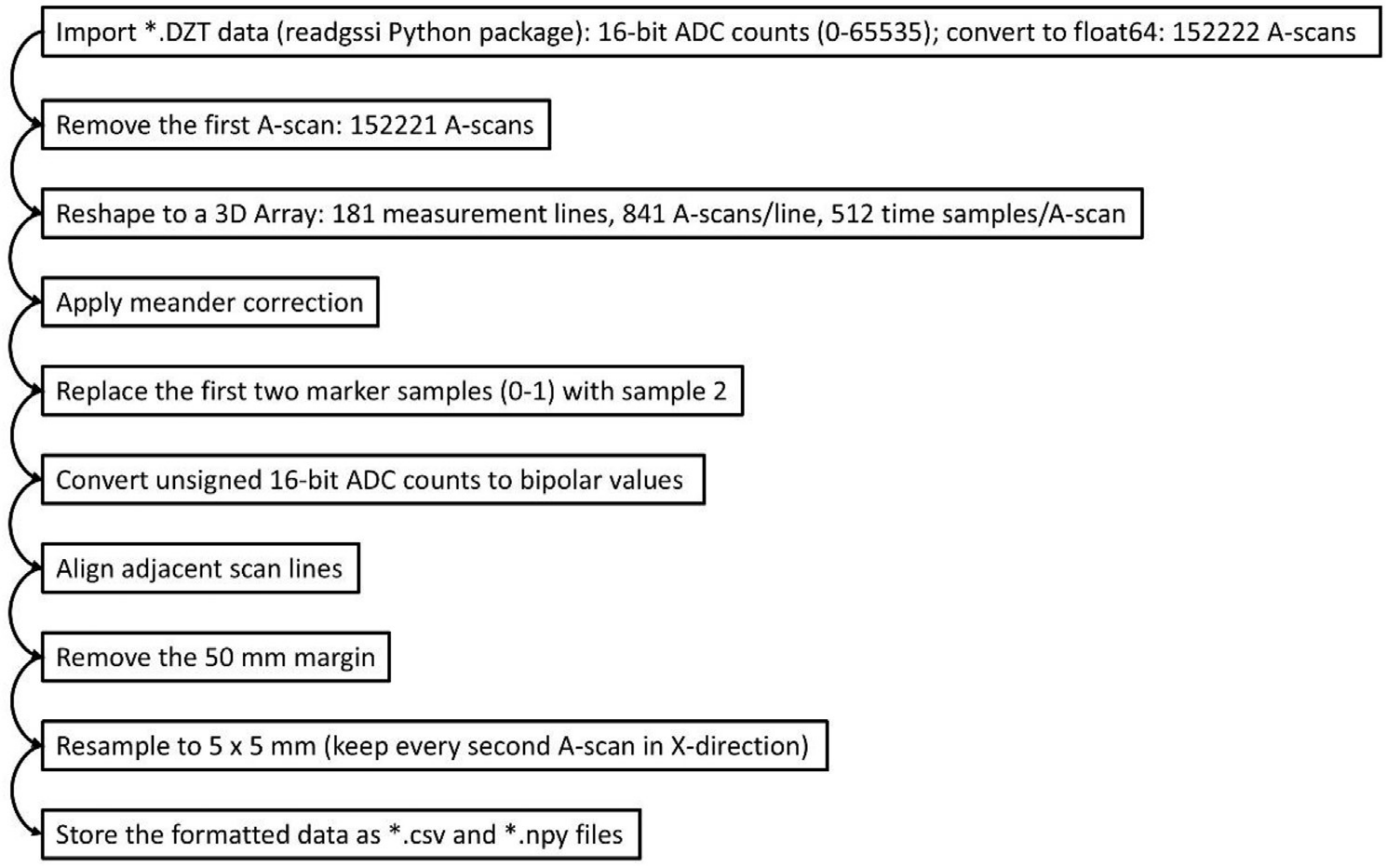
Workflow of the data formatting steps applied to the original radar files in the *.DZT format.

**Fig. 13:**
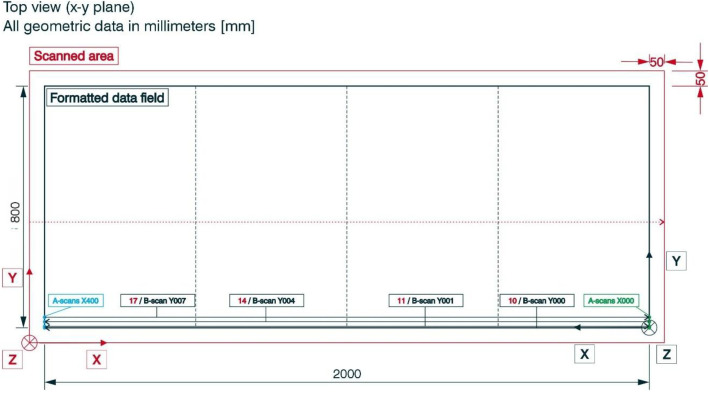
Overview of the final formatted data field. The example scan lines No. 11, No. 14, and No. 17 from Fig. 11 correspond to B-scans Y001, Y004, and Y007 in the formatted dataset. Scan line No. 10 corresponds to B-scan Y000. The first and last A-scans of each B-scan are marked and labelled with their dataset designations, X000 and X400.

The workflow shown in Fig. 12 was implemented in Python (Python version 3.10.18) to format the native *.DZT files from the SIR-20 system into *.npy and *.csv datasets. This step was necessary because the SIR-20 system stores the recorded A-scans in one continuous line, regardless of the scanning grid (152222 A-scans in total). First, the ADC counts were imported using the readgssi package and converted to float64 to enable further processing. The first A-scan was then removed, as it represented a redundant data point produced by the radar unit. The data were reshaped into a 3D array with 181 measurement lines, 841 A-scans per line, and 512 time samples per A-scan (Table 3). To correct the meandering acquisition, every second scan line was reversed along the X-direction. The reversal started with the first scan line (No. 0) to ensure that the coordinate origin matches the origin that was used by Maack et al. [1] and Grohmann et al. [2] for the ultrasonic measurements (black crossed circle in Fig. 13 and APPENDICES A - C). In addition, the first two samples of each A-scan, which contain system-internal marker information rather than measurement data, were replaced with the third sample. The unsigned 16-bit ADC counts were then converted to signed values to obtain a bipolar signal representation. A spatial alignment between adjacent scan lines was applied to compensate for the offset between even and odd lines (details are given in section LIMITATIONS). Afterwards, the measurement points outside the specimen were removed, resulting in a final data field with a length of X = 2000 mm and a width of Y = 800 mm (marked black in Fig. 13). Finally, to obtain a uniform 5 × 5 mm grid, every second A-scan in the X-direction was kept. No interpolation was used. To address the spatial sampling criterion, the final 5 × 5 mm grid was checked using the highest recorded frequency of 5 GHz (Table 2) and the lowest estimated apparent wave velocity (Table 4). With $v_{app}$= 0.1088 m/ns and $f_{max}$ = 5 GHz, the quarter-wavelength criterion $\Delta x\leq \;\frac{v_{app}}{4f_{max}}$ gives a maximum spatial sampling interval of 5.44 mm. Therefore, the final 5 mm × 5 mm grid is adequate for spatial sampling of the recorded GPR signals.

**Table 3:** Comparison of the scanned area and the formatted data field for Pk050, Pk266 and Pk401.

Parameter	Scanned area	Formatted data field
Area size [X x Y] [mm]	2100×900	2000×800
Grid spacing [X x Y] [mm]	2.5 × 5	5 × 5
Number of measurement points X-direction []	841	401
Number of scan lines Y-direction []	181	161
3D array dimensions [X × Y × time samples]	841 × 181 × 512	401 × 161 × 512
Sampling density X-direction [Samples/cm]	4	2
Sampling density Y-direction [Samples/cm]	2	2

**Table 4:** Apparent electromagnetic wave velocity, apparent relative permittivity, and time-zero correction estimated from aluminium back-wall reflections for the 1.5 GHz antenna and Rot00 orientation.

Specimen	Antenna type & orientation	$v_{app}$[m/ns]	$t_{0}$ [ns]	$\varepsilon_{r,app}$ []
Pk266	1.5 GHz & Rot00	0.1271	0.7308	5.56
Pk050	1.5 GHz & Rot00	0.1246	0.6723	5.79
Pk401	1.5 GHz & Rot00	0.1088	0.6868	7.6

The resulting formatted datasets were stored as *.npy and *.csv files. The spatial parameters before and after data formatting are summarized in Table 3. Fig. 13 shows the scan directions, and dataset designations of the example scan lines No. 11, No. 14, and No. 17 from Fig. 11 after data formatting. In the formatted datasets, these scan lines correspond to B-scans Y001, Y004, and Y007, respectively. Scan line No. 10 corresponds to B-scan Y000. Fig. 13 also indicates the positions of the first and last A-scans of each B-scan in the formatted datasets, denoted as X000 and X400 (see Fig. 3, Fig. 6 in section DATA DESCRIPTION).

### 4.5 Reference Information For Data Use

As reference information for dataset use, an apparent electromagnetic wave velocity $v_{app}$ was estimated from the GPR data. These values were estimated from the aluminium back-wall reflections at known specimen thicknesses d, which provide well-defined reference events for velocity estimation $v_{app}$ and time-zero correction $t_{0}$. A monostatic approximation of the antenna configuration was used, according to $t_{BW}=\;t_{0}+\frac{2d}{v_{app}}$. For each concrete specimen and the dataset acquired with the 1.5 GHz antenna (orientation of antenna: Rot00), back-wall reflection times $t_{BW}$ were picked in the four thickness steps. For each thickness step, six back-wall reflection times were picked from six different B-scans and then averaged. The mean back-wall reflection times were then related to the known specimen thicknesses d by a linear fit. From this fit, the apparent wave velocity $v_{app}$ and an effective time-zero correction $t_{0}$ were estimated. The corresponding apparent relative permittivity was calculated from the estimated apparent velocity using $\varepsilon_{r,app}={\left(\frac{c}{v_{app}}\right)}^{2}$, where c is the speed of light in vacuum. The resulting values for all three concrete specimens and the 1.5 GHz antenna are reported in Table 4. These values should be understood as specimen-specific apparent estimates which can be used for approximate time-to-depth conversion and not as independently measured material properties. For a picked reflection time t, the corresponding depth d can be estimated as $d=\;\frac{v_{\mathrm{app}}\;\left(t-t_{0}\right)}{2}$.

The differences between the specimens shown in Table 4 are expected, although the available material parameters show the same concrete mixture for Pk050 and Pk266, as well as a similar concrete mixture for Pk401 (Table 1). The effective electromagnetic properties of concrete can be influenced by local aggregate distribution, porosity, compaction, hardening and storage conditions, as well as moisture state. For example, Pk401 was manufactured separately from Pk050 and Pk266 and was stored under different conditions, which may have influenced its apparent electromagnetic properties (section CONCRETE SPECIMENS PK050, PK266, AND PK401). Therefore, similar mixture parameters and the same aggregate grading curve (Table 1) do not necessarily result in identical apparent wave velocities in the specimens. The specimens should therefore be understood as well-documented laboratory specimens rather than exact reference materials. In addition, other picking criteria or processing steps may lead to slightly different velocity estimates. Dataset users can use the reported values as reference information or repeat the estimation with their own picking and processing approach.

**Fig. 14:**
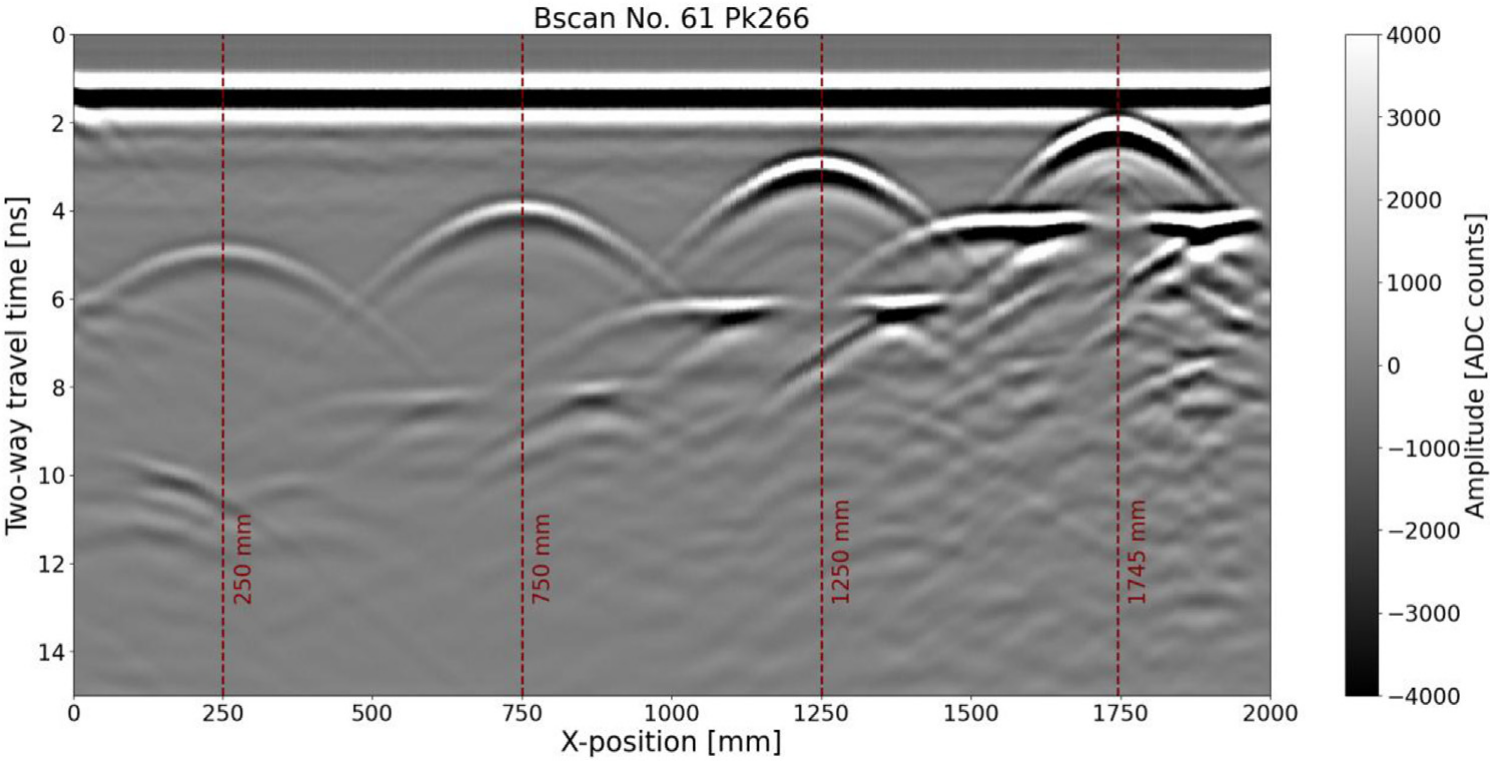
Example B-scan from specimen Pk266 with marked lateral positions of the tendon ducts. The positions were identified from the apices of the reflection hyperbolas in the B-scan.

As an additional reference for dataset use, Fig. 14 shows an example B-scan from specimen Pk266. The lateral positions of the four tendon ducts were identified from the apices of the reflection hyperbolas and are marked with vertical dashed lines. The identified positions are consistent with the documented specimen geometry (APPENDIX B). For the tendon duct documented at X = 1750 mm, the identified lateral position is X = 1745 mm, resulting in a difference of 5 mm. This difference corresponds to one grid interval of the formatted dataset and is therefore within the spatial resolution of the provided 5 mm × 5 mm data. This example should be understood as a plausibility check rather than as an independent validation of the exact lateral positions of the tendon ducts.

## Limitations

After the meander correction applied within the workflow presented in Fig. 12, a small spatial offset was observed between even and odd scan lines. This offset is related to the measurement setup: two rotary encoders on the scanner axes provide the position information from which the scanner control generates the trigger pulses for the SIR-20 system. Because there is a delay between the external trigger and the actual start of the data acquisition in the SIR-20 system, the recorded A-scans are slightly shifted in the measurement direction (X-direction). This in turn causes a displacement between neighbouring scan lines. To compensate for this effect, the scan lines were aligned with each other (see Fig. 12). Even-numbered scan lines (starting with scan line No. 0) were shifted in one direction and odd-numbered scan lines in the opposite direction. The correction was done by inserting zero traces on one side of the B-scan and cutting the same number of traces on the other side (even lines: zeros at the end; odd lines: zeros at the beginning). Between 3 and 6 zero traces were inserted, depending on the dataset (Table 5). These zero traces were introduced only at the outer edge of the scanned area and lay within the margin region (50 mm margin; Fig. 11) that was removed during data formatting (Fig. 12). In this way, the spatial offset caused by the measurement setup was corrected without changing the overall dimensions of the data. A small residual misalignment may remain due to the discrete nature of the applied shifts but is expected to be smaller than half a trace spacing (< 1.25 mm) and therefore negligible.

**Table 5:** Number of inserted traces used for scan line alignment (with the first scan line defined as index 0).

Specimen	Antenna type [GHz]	Orientation	Zero traces	
			Odd scan lines	Even scan lines
Pk050	1.5	Rot00	5	4
		Rot90	4	4
	2.6	Rot00	5	5
		Rot90	4	3
Pk266	1.5	Rot00	4	5
		Rot90	4	3
	2.6	Rot00	4	5
		Rot90	4	4
Pk401	1.5	Rot00	5	5
		Rot90	5	6
	2.6	Rot00	5	5
		Rot90	4	4

During data acquisition in a meander pattern, the antenna moved continuously in the measurement direction (X-direction). Since the antenna is moving while an A-scan is being recorded, the 512 samples within one A-scan come from slightly different positions rather than from a single fixed measurement point. This causes a small “smearing” of the A-scan along the measurement direction and can slightly broaden reflections in the B-scan. Because the measurement direction alternates from one line to the next in a meander pattern, the smearing appears in opposite directions in neighbouring scan lines. Given the measurement point spacing of 2.5 mm, the resulting smearing is expected to be only marginally observable in the data.

## Ethics Statement

The authors have read and follow the ethical requirements for publication in Data in Brief, and confirm that the data do not involve human subjects, animal experiments, or any data collected from social media platforms.

## CRediT Author Statement

**Maria Grohmann:** Conceptualization, Methodology, Investigation, Software, Formal analysis, Writing – original draft, Data curation; **Jens Wöstmann:** Methodology, Investigation; **Stefan Maack:** Methodology, Formal analysis, Writing – review & editing; **Ernst Niederleithinger:** Supervision, Writing – review & editing.

## Data Availability

DataverseGround-Penetrating Radar Data (Pulse-Echo Mode) Acquired with 1.5 GHz and 2.6 GHz Antennas on Concrete Step Specimens Containing Embedded Objects (Original data).

## Appendices

APPENDIX A: TECHNICAL DRAWINGS OF PK050.

APPENDIX B: TECHNICAL DRAWINGS OF PK266.

APPENDIX C: TECHNICAL DRAWINGS OF PK401 (Reproduced from [2]).
